# AI prediction models based on time-lapse imaging for good embryos with implantation potential and euploidy

**DOI:** 10.1038/s41598-026-40917-5

**Published:** 2026-02-19

**Authors:** Ryo Maekawa, Taro Kiritani, Takeshi Abe, Masahiko Nakatsui, Yoshiyuki Asai, Norihiro Sugino

**Affiliations:** 1 Department of Obstetrics and Gynecology, Yamaguchi University Graduate School of Medicine, Minami Kogushi 1-1-1, Ube, Japan; 2 Department of Obstetrics and Gynecology, Nara Medical University, Kashihara, Japan; 3 Exawizards Co. Ltd, Tokyo, Japan; 4 AI Systems Medicine Research and Training Center, Yamaguchi University Graduate School of Medicine, Yamaguchi, Japan; 5 Division of Systems Medicine and Informatics, Research Institute for Cell Design Medical Science, Yamaguchi University Graduate School of Medicine, Yamaguchi, Japan; 6 Department of System Bioinformatics, Yamaguchi University Graduate School of Medicine, Yamaguchi, Japan

**Keywords:** Embryo selection, Deep learning, Time-lapse imaging, Clinical pregnancy, PGT-A, Artificial intelligence, Computational biology and bioinformatics, Developmental biology, Medical research

## Abstract

Embryo selection in assisted reproduction has traditionally relied on subjective morphological grading, which is prone to inter-observer variability and correlates poorly with ploidy, the primary cause of miscarriage. Artificial intelligence (AI) models utilizing time-lapse imaging have emerged; however, many rely on human-defined morphokinetic markers or static blastocyst images. We developed two deep learning models that non-invasively predict the likelihood of clinical pregnancy and embryo ploidy status, based solely on time-lapse videos and maternal age. In this retrospective study, 2,436 embryos were analyzed for pregnancy prediction and 1,645 for ploidy prediction, with external validation performed using data from independent IVF centers. Both models were implemented using a spatiotemporal convolutional neural network. The pregnancy prediction model achieved an area under the receiver operating characteristic curve (AUROC) of 0.799 in the validation set, 0.717 in the internal test set, and 0.746 in the external test set. The ploidy prediction model yielded AUROCs of 0.802, 0.738, and 0.759 in the validation, internal, and external test sets, respectively. These results suggest that AI models can serve as non-invasive decision support tools that leverage developmental dynamics associated with implantation potential and chromosomal status. These models may supplement conventional morphology-based assessment and contribute to more objective embryo selection.

**Supplementary Information:**

The online version contains supplementary material available at 10.1038/s41598-026-40917-5.

## Introduction

The selection of viable embryos for transfer remains a critical challenge in assisted reproductive technology (ART). Traditionally, embryo selection has relied on morphological assessments such as the Gardner classification, which evaluates blastocyst expansion, inner cell mass, and trophectoderm quality^1,2^. Although widely adopted, this method is inherently subjective, as it depends on the visual judgment of embryologists, and is therefore prone to inter-observer and intra-observer variability. This variability means that different embryologists may prioritize different embryos for transfer, leading to inconsistent decisions and ultimately less predictable treatment outcomes^3,4^.

Another primary consideration in embryo selection is the chromosomal status of the embryo. Euploidy, defined as a normal chromosomal complement, is strongly associated with successful embryo development, whereas aneuploidy leads to implantation failure or miscarriage. Although morphological grading by the Gardner classification reflects some association with ploidy status, the correlation remains weak. Studies have shown that a significant portion of morphologically high-grade embryos remains aneuploid, and overall morphology cannot reliably distinguish chromosomal normality^5^. Preimplantation genetic testing for aneuploidy (PGT-A) offers a direct evaluation of chromosomal normality and can enhance the success of embryo transfers. However, it involves an invasive biopsy procedure, which raises concerns about potential harm to embryo viability and the long-term health of the offspring^6–8^. Consequently, developing a non-invasive method capable of reliably assessing embryo ploidy would be of great clinical value for embryo selection.

In recent years, deep learning models using time-lapse embryo imaging have been developed for both pregnancy prediction and ploidy status assessment. Previous studies have reported convolutional neural networks that predict implantation outcomes directly from embryo videos, as well as approaches that infer chromosomal status using single static images^9–12^. These models have demonstrated improved predictive accuracy compared to conventional morphology-based methods, but some still rely on human-defined inputs such as morphokinetic markers or Gardner classification scores^13–16^. Notably, previous studies have adopted different strategies for handling non-transferred embryos in pregnancy prediction tasks, including treating them as non-pregnant cases during model development^17^. Such strategies may be reasonable depending on the intended application. However, because implantation outcomes cannot be directly observed in embryos that were not transferred, pregnancy labels in the present study were assigned exclusively to transferred embryos with clearly defined gestational outcomes.

Time-lapse imaging captures continuous information on embryo development, including subtle morphological changes and the timing of cell divisions that are often imperceptible to the human eye. While many earlier approaches focused on manually selected morphokinetic time points^14–16^, more recent work has increasingly adopted end-to-end deep learning on embryo images or time-lapse sequences, and commercially available AI-based embryo scoring tools have also been introduced (e.g., ERICA; iDAScore)^9,18^. The importance of dynamic information across the entire culture period has been emphasized in consensus guidelines^3,4^ and recent reviews^19^, supporting the potential value of models that can exploit the complete video stream. We hypothesize that bypassing human-defined feature selection and allowing the model to learn directly from the complete video stream could better capture biologically relevant signals related to implantation and chromosomal status.

To overcome these limitations, we developed two deep learning-based models that use only time-lapse embryo videos and maternal age, independent of manual grading or predefined morphokinetic markers. The Clinical Pregnancy Prediction Model was trained to estimate the likelihood of establishing a clinical pregnancy, while the Ploidy Prediction Model was designed to distinguish euploid from aneuploid embryos in the primary analysis. Both models employed an ensemble prediction strategy, combining predictions from multiple networks to improve robustness.

## Results

### Model performance across time steps

To examine how different stages of embryo development contribute to model performance, we analyzed predictive accuracy across multiple discrete time points and cumulative developmental windows. The Clinical Pregnancy Prediction Model was evaluated from 20 h to 90 h, as well as at cumulative windows up to 50 h, to identify which developmental stages contributed most to predictive accuracy. Table 1 reports the mean ± SD AUROC across ten independently trained models for each time point and cumulative window. Predictive performance showed a gradual decline from approximately 20 to 90 h, suggesting that earlier developmental events have relatively greater predictive value. Limiting the input to data up to the 50-hour window (AUROC = 0.774 ± 0.007) yielded higher accuracy than using the entire culture period (AUROC = 0.764 ± 0.012).

**Table 1:** Mean and standard deviation of AUROC for each discrete time point and cumulative time window in the clinical pregnancy prediction model (validation dataset).

Time point or cumulative window	Mean AUROC	SD of AUROC
20 h	0.761	0.012
30 h	0.749	0.014
40 h	0.739	0.016
50 h	0.736	0.022
60 h	0.732	0.026
70 h	0.725	0.023
80 h	0.722	0.02
90 h	0.702	0.011
Up to 50 h	0.774	0.007
Entire culture period	0.764	0.012

AUROC: Area under the receiver operating characteristic curve.

“Entire” uses the full culture period.

Values are mean ± SD of AUROC across ten independently trained single models (different random seeds).

The Ploidy Prediction Model was similarly evaluated across discrete time points and cumulative windows. Table 2 reports the mean ± SD AUROC across ten independently trained models for each time point and cumulative window. In contrast to the Clinical Pregnancy Prediction Model, predictive performance in this model remained relatively stable across the culture period. The highest accuracy was observed when using the whole culture period (AUROC = 0.802 ± 0.008). Time-point–specific and cumulative performance analyses for both the clinical pregnancy and ploidy prediction tasks across the validation, internal test, and external test datasets are provided in Supplementary Table S1.

**Table 2:** Mean and standard deviation of AUROC for each discrete time point and cumulative time window in the ploidy prediction model (validation dataset).

Time point or cumulative window	Mean AUROC	SD of AUROC
20 h	0.794	0.017
30 h	0.78	0.015
40 h	0.776	0.015
50 h	0.766	0.009
60 h	0.787	0.018
70 h	0.783	0.025
80 h	0.779	0.019
90 h	0.766	0.022
Up to 50 h	0.793	0.011
Entire culture period	0.802	0.008

AUROC: Area Under the Receiver Operating Characteristic curve.

“Entire” uses the full culture period.

Values are mean ± SD of AUROC across ten independently trained single models (different random seeds).

### Score normalization before ensemble aggregation

To increase robustness, we constructed an ensemble by aggregating predictions from ten independently trained models, allowing the final output to reflect a consensus across models rather than depending on a single network. This strategy reduces the impact of model-specific variability and provides more stable predictions. However, the raw prediction scores differed in scale across models (Supplementary Fig. S1), which could otherwise bias the ensemble toward certain models. To standardize the outputs across models, we applied robust z-score normalization, a statistical normalization step that aligns score distributions across models, using parameters summarized in Supplementary Tables S2 and S3, and illustrated in Supplementary Figure S1. This adjustment enabled valid aggregation and prevented models giving consistently larger or smaller scores from unfairly outweighing the others, regardless of their actual accuracy. An overview of this process is provided in Supplementary Figure S4.

### Ensemble model performance

**Fig. 1:**
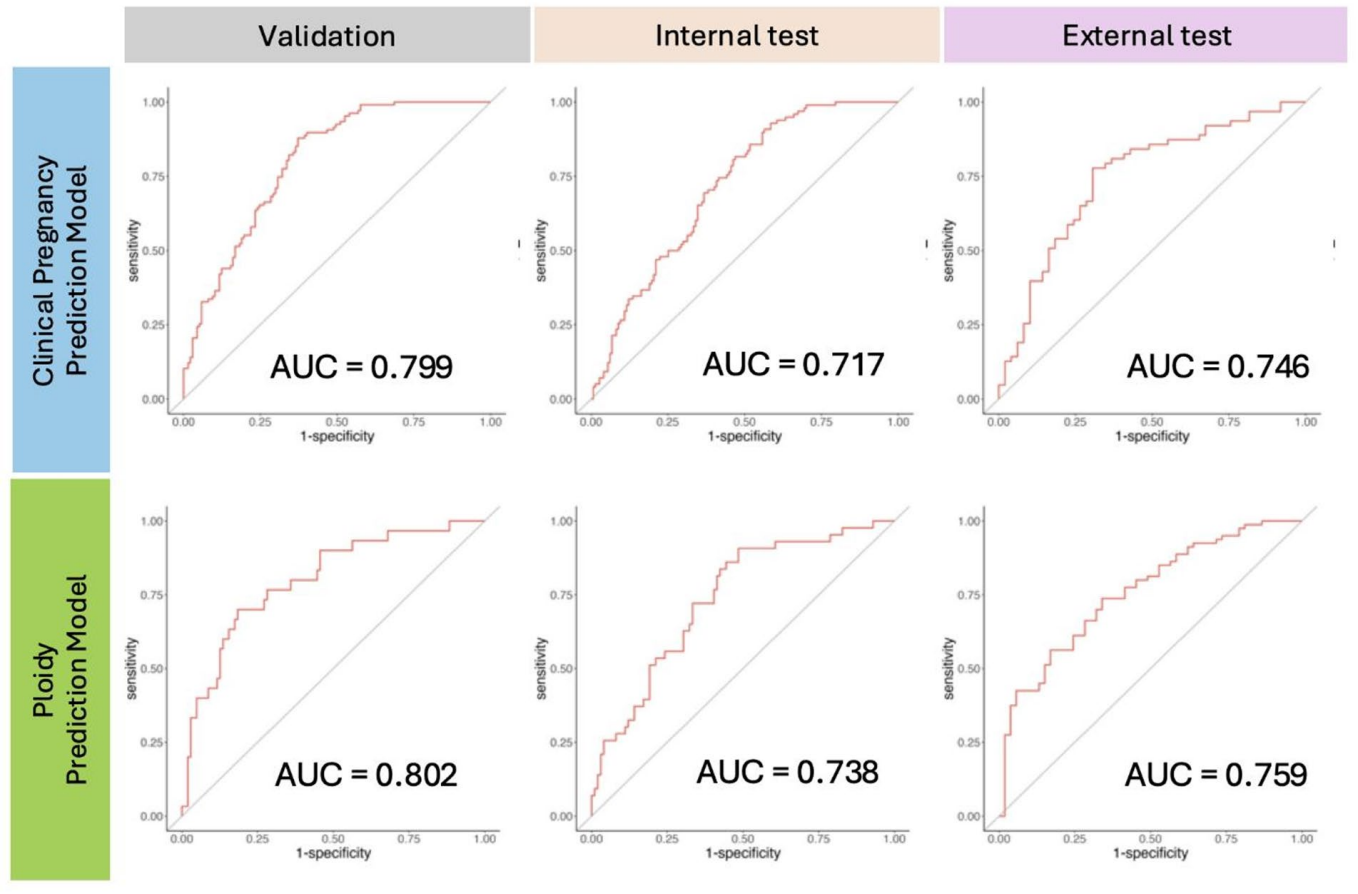
Ensemble model performance in validation, internal test, and external test datasets. Receiver operating characteristic (ROC) curves of the Clinical Pregnancy Prediction Model (top row) and the Ploidy Prediction Model (bottom row) after robust z-score normalization and ensemble aggregation. The Clinical Pregnancy Prediction Model was evaluated at the U50 cumulative window (20–50 h), whereas the Ploidy Prediction Model was evaluated across the entire culture period, which provided the best overall performance.

We next evaluated the overall performance of the ensemble models. The Clinical Pregnancy Prediction Model was evaluated at the 20–50 h cumulative window (U50), which yielded the highest AUROC in the time-window analysis. In the validation dataset, the model had an AUROC of 0.799 (Fig. 1). In the internal test dataset, the model had an AUROC of 0.717, while in the external test dataset, the AUROC was 0.746. To assess the contribution of image-based features beyond baseline clinical variables, we additionally compared the ensemble model with a logistic regression model using only maternal age. As summarized in Supplemental Table S4, the age-only model showed substantially lower AUROC values across the validation, internal test, and external test datasets, whereas the age-plus-image model consistently achieved higher performance.

The Ploidy Prediction Model was evaluated using the whole culture period, which provided the best overall performance. In the validation dataset, the model had an AUROC of 0.802 (Fig. 1). In the internal and external test datasets, the model had AUROCs of 0.738 and 0.759, respectively. A similar comparison was performed for the ploidy prediction task, demonstrating that incorporation of time-lapse image features showed a trend toward higher discrimination compared with age-only models across all datasets, although the confidence intervals overlapped (Supplemental Table S4).

These results indicate that both models maintained consistent predictive ability across datasets, with broadly comparable performance between the two models. For completeness, the performance of a baseline model incorporating maternal age and temporal information without image features is provided in Supplementary Table S4.

### Performance at clinically optimized thresholds

**Fig. 2:**
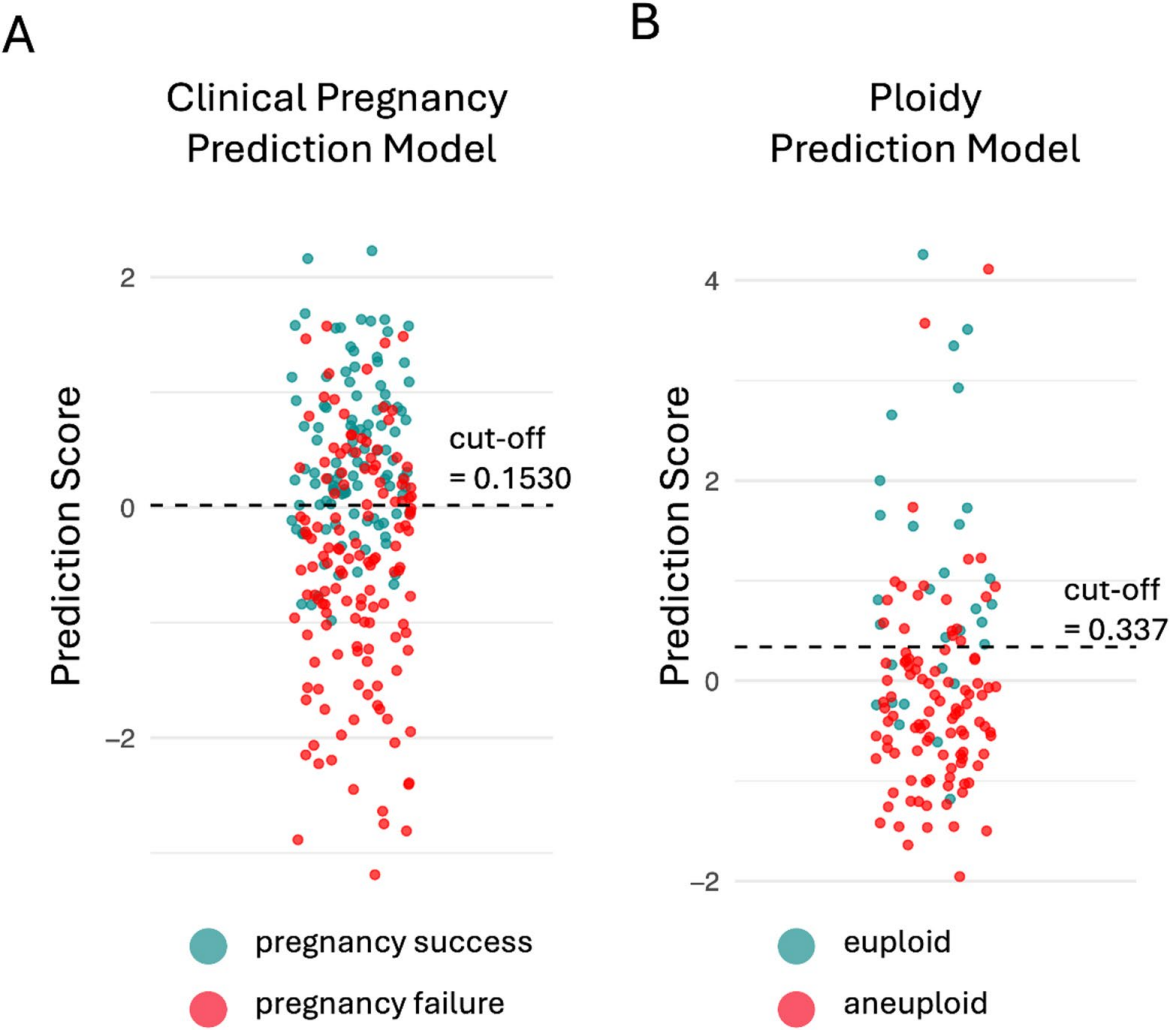
Distribution of model prediction scores for embryos in the validation dataset. (**A**) Clinical pregnancy prediction model. (**B**) Ploidy prediction model. Each dot represents an embryo, with cyan indicating successful pregnancies or euploid embryos and red indicating unsuccessful pregnancies or aneuploid embryos. The dashed line indicates the ROC-derived cut-off selected by the “topleft” criterion, optimized on the validation dataset.

While AUROC summarizes ranking performance, it does not indicate how many embryos would be recommended for transfer or withheld at a given operating point. Clinical use requires selecting a decision threshold that balances sensitivity (retaining embryos with implantation potential or euploid status) and specificity (excluding embryos with poor prognosis or aneuploid status). We therefore performed threshold-based performance evaluation, using thresholds determined by the ROC “topleft” criterion in the validation dataset, and applied the same fixed thresholds to the internal and external test sets to approximate real-world deployment and assess generalizability (Fig. 2). For the Clinical Pregnancy Prediction Model (cut-off = 0.1530), the validation set retained 77.8% of embryos that resulted in clinical pregnancy while excluding 69.4% of embryos that failed to implant, yielding an accuracy of 74.1% (Table 3). When applied to the internal test set, the same threshold achieved a sensitivity of 74.5% and a specificity of 58.5%, yielding an accuracy of 64.9%. In the external test set, sensitivity was 80.4% and specificity was 66.4%, resulting in an overall accuracy of 72.5%. Clinically, at this fixed operating point, these findings suggest that the model may preserve most embryos with implantation potential while excluding approximately two-thirds of embryos with poor prognosis, supporting its potential use for prioritization rather than definitive embryo selection.

**Table 3:** Performance metrics of ensemble models at optimized clinical thresholds in validation, internal test, and external test datasets.

Dataset	Sensitivity	Specificity	PPV	NPV	Accuracy
Clinical pregnancy prediction model					
Validation	0.778	0.694	0.766	0.708	0.741
Internal test	0.745	0.585	0.545	0.775	0.649
External test	0.804	0.664	0.652	0.813	0.725
Ploidy prediction model					
Validation	0.7	0.816	0.525	0.903	0.789
Internal test	0.628	0.697	0.474	0.812	0.676
External test	0.813	0.509	0.714	0.643	0.692

The cut-off value was determined in the validation dataset using the ROC criterion that minimizes the distance to the upper-left corner (0,1) and was applied unchanged to the internal test and external test datasets. The resulting cut-off values were 0.153 for the Clinical Pregnancy Prediction Model and 0.337 for the Ploidy Prediction Model.

For the Ploidy Prediction Model (cut-off = 0.337), the validation set retained 70.0% of euploid embryos while correctly excluding 81.6% of aneuploid embryos, yielding an accuracy of 78.9% (Table 3). When applied to the internal test set, the same threshold achieved a sensitivity of 62.8% and a specificity of 69.7%, yielding an accuracy of 67.6%. In the external test set, the same threshold retained 81.3% of euploid embryos and excluded 50.9% of aneuploid embryos, with an accuracy of 69.2%. Clinically, these results suggest that the model may prioritize embryos predicted to be euploid while limiting the inclusion of likely aneuploid embryos at the predefined operating point.

### Age-stratified model performance and calibration

To evaluate whether the models can be used for embryo ranking within age-homogeneous treatment cycles, we performed age-stratified analyses according to standard SART age groups. Discriminative performance (AUROC) of the age-only and age-plus-image models for clinical pregnancy prediction and ploidy prediction is summarized in Supplementary Tables S5 and S6, respectively. Across age strata, AUROC estimates were associated with wide confidence intervals due to limited sample sizes; however, the age-plus-image model did not collapse to age-only performance within age-constant cohorts, supporting its utility beyond age-related effects. Model calibration was evaluated using reliability plots across the entire cohort for both prediction tasks (Supplementary Figure S2 and S3). Age-stratified calibration analysis was not performed because stratification substantially reduced the number of samples per bin, precluding reliable estimation. The overall calibration results indicate reasonable agreement between predicted probabilities and observed outcomes.

## Discussion

A pivotal strength of our model lies in its ability to learn directly from the entire time-lapse video sequence, rather than from preselected static images or manually defined features. This comprehensive use of temporal and morphological information enabled robust, high-accuracy prediction of both clinical pregnancy and ploidy status, offering improvements over traditional morphology-based assessments. Notably, the performance of our approach is comparable to that reported in representative non-invasive embryo assessment models based on time-lapse imaging or static blastocyst images^20,21^, emphasizing the value of leveraging the dynamic information embedded in embryo videos. Moreover, the ensemble framework provided stable performance across validation, internal, and external datasets. Together, these results show that a fully data-driven, ensemble-based strategy can deliver robust and generalizable performance. This approach may help overcome the limitations of morphology-based or marker-dependent embryo assessment and lay a foundation for more objective, non-invasive embryo selection in ART.

Although our study was not designed to explicitly identify individual morphological biomarkers, several aspects of our framework support the biological relevance of the learned representations. By operating directly on the complete time-lapse video stream and avoiding human-defined morphokinetic markers or grading scores, the model was allowed to learn developmental patterns directly from raw embryo dynamics. In addition, the consistency of predictive performance across internal and external cohorts argues against reliance on center-specific artifacts. Nevertheless, we acknowledge that explicit interpretability of the spatiotemporal features underlying model predictions was beyond the scope of the present study. Future work incorporating visualization and explainability methods may help clarify which developmental regions and time windows contribute most strongly to prediction.

Another strength of our framework lies in its ensemble design. By training ten independent models and aggregating their outputs through a statistical normalization step, the final decision reflects a consensus rather than depending on a single model. This strategy approximates the consensus decision-making process of multiple embryologists, yielding more stable and reproducible results. Importantly, the ensemble aggregation strategy was designed to reduce model-to-model variability and improve robustness to data heterogeneity and institutional variability. Such resilience is critical for clinical translation, where variability in imaging conditions and patient populations is unavoidable. Another strength lies in the dedicated preprocessing pipeline, optimized for time-lapse data, which includes dynamic background suppression, fixed magnification to a standardized spatial scale, and automated centering. These steps standardized the images and minimized distractions from debris, lighting fluctuations, or embryo drift. This helped the network focused on biologically meaningful features, improving the reliability of its representations.

Several AI-based embryo assessment systems have been developed in recent years, many of which rely on a single static blastocyst image, typically captured around day 5, to predict ploidy or implantation potential^9,11,12^. Because these approaches are based on one-time images, they cannot capture dynamic developmental events such as cleavage timing, compaction, and blastulation—events highlighted in the Istanbul consensus as critical to embryo evaluation^3,4^. The value of time-resolved data is increasingly recognized. Recent expert reviews emphasize that time-lapse imaging should be leveraged not only for manual annotation but also for AI-based modeling, enabling the full use of dynamic information without the preselection of specific frames^4^. Commercial systems, such as iDAScore^18^, represent progress toward automated scoring; however, they still emphasize later developmental stages and may underexploit early events. Similarly, a recent deep learning framework predicted ploidy from time-lapse videos but focused mainly on blastocyst-stage frames, leaving earlier windows insufficiently examined^21^.

In contrast to these approaches, our model systematically profiled multiple time points and cumulative windows. We found that the prediction of clinical pregnancy benefited most from early developmental windows. In contrast, ploidy prediction remained stable throughout the entire culture period, exhibiting similar accuracy across both early and later developmental stages. This finding is consistent with prior reports highlighting the importance of early cleavage dynamics: embryos reaching the 2-cell stage within ~ 25 h showed higher blastocyst formation rates^22^, early cleavage timing correlated with better morphology and implantation outcomes^23^, and recent reviews have reinforced the predictive value of early cell division intervals such as the 3- to 4-cell transition^24^. A broader synthesis has further emphasized the associations between parameters such as t2 (time to 2-cell), t4 (time to 4-cell), and s2 (the interval between them) with implantation and chromosomal normality, supporting the biological plausibility that early developmental windows carry strong prognostic signals^19^. We acknowledge that this observation differs from conclusions of prior morphokinetic-based studies, including the meta-analysis by Bamford et al. which emphasized later-stage morphokinetic parameters^25^. Most studies included in that meta-analysis relied on predefined morphokinetic annotations or manually selected timing variables. In contrast, our analysis was based on end-to-end learning from raw time-lapse video data, and the observed difference likely reflects methodological distinctions between the two approaches. Observed variability in performance across internal and external cohorts may reflect inter-center differences rather than substantial model instability, given that the relative contribution of image-based features remained consistent across datasets.

Both models enhance traditional morphology assessments and other non-invasive evaluations, improving embryo transfer decisions and optimizing resource use. The Clinical Pregnancy Prediction Model can help reduce the time to pregnancy by enabling efficient embryo prioritization, thereby alleviating the physical and emotional burden on patients. The Ploidy Prediction Model provides a non-invasive indicator for chromosomal status and may complement or, where PGT-A is not feasible, partially substitute for PGT-A. By providing non-invasive estimates of ploidy status, this model has the potential to reduce miscarriage rates and lessen reliance on PGT-A. It may also lower treatment costs and minimize the risks associated with invasive procedures. In addition, it can serve as a pre-screening tool to optimize the application of PGT-A.

From a clinical perspective, the proposed models are intended to function as decision-support tools rather than as autonomous decision-makers. They can be integrated into existing IVF laboratory workflows by analyzing time-lapse videos that are already routinely acquired during embryo culture, without requiring additional procedures or changes to standard clinical practice. In practice, prediction scores from the Clinical Pregnancy Prediction Model may be used to prioritize embryos for transfer within a cohort, particularly when multiple embryos are available and morphological assessment alone yields ambiguous rankings. Similarly, the Ploidy Prediction Model may serve as a non-invasive pre-screening tool to support embryo prioritization or the selective application of PGT-A, especially in settings where invasive testing is not feasible or routinely performed. Importantly, model outputs are designed to complement—rather than replace—embryologists’ assessments and clinical judgment, with final transfer decisions remaining under the responsibility of clinicians who integrate model predictions with conventional morphology, patient-specific factors, and the overall clinical context.

This study has several limitations. First, unmeasured inter-facility factors—such as culture medium, gas concentrations, and procedural variations—may influence predictions. Additionally, all data in this study were derived from EmbryoScope systems, which may limit the generalizability of the findings to other time-lapse platforms. Multicenter validation under diverse laboratory conditions will help address these issues. Second, our datasets were derived exclusively from Japanese IVF populations; testing in international, multiethnic cohorts will be essential to establish broader generalizability. Third, regarding pregnancy prediction, implantation, and pregnancy outcomes are also influenced by maternal factors such as endometrial receptivity and uterine environment, which were not captured in this study. Finally, the study was retrospective, underscoring the need for prospective, double-anonymized trials to confirm whether the model improves outcomes beyond morphology-based selection.

Although our framework was intentionally designed to be image-based, further improvements could come from integrating routinely collected clinical variables, such as anti-Müllerian hormone (AMH) levels or prior IVF history, to capture the interplay between patient-specific factors and embryo dynamics. Another important direction is improving explainability. Developing spatiotemporal visualization tools to highlight which developmental regions and time windows drive predictions could increase transparency, foster embryologists’ trust, and facilitate integration into routine IVF practice. Recent work by Rotem et al. has already demonstrated the feasibility of such visualization approaches in embryo assessment, underscoring their potential clinical value^26^.

In summary, we developed and externally validated two complementary AI models that leverage time-lapse imaging to predict embryo implantation potential and chromosomal normality non-invasively. By learning directly from the complete time-lapse video sequence and integrating predictions through an ensemble framework, our models achieved robust and generalizable performance across datasets. Together, these findings demonstrate how unbiased, time-aware AI evaluation of embryo development can overcome the significant limitations of morphology-based assessment and PGT-A, thereby paving the way toward more effective, equitable, and non-invasive embryo selection in assisted reproductive technology.

## Materials and methods

### Data acquisition

This study was approved by the Ethics Committee of the Faculty of Medicine and University Hospital, Yamaguchi University (approval number: 2020 − 101). We retrospectively collected data from two independent IVF centers in Japan between January 2019 and December 2021. All time-lapse imaging data were acquired using the EmbryoScope^®^ time-lapse incubator system (Vitrolife, Denmark), ensuring standardized imaging conditions across institutions. For the Clinical Pregnancy Prediction Model, data from 2,436 embryos were used; for the Ploidy Prediction Model, data from 1,645 embryos were analyzed. Each dataset comprised time-lapse imaging data and the maternal age at the time of oocyte retrieval. Embryos were included if complete time-lapse recordings from fertilization to transfer or biopsy were available, along with documented maternal age and clinical outcome. Embryos with incomplete imaging data or missing outcome information were excluded. Clinical pregnancy was defined as sonographic confirmation of a gestational sac. PGT-A results were categorized as euploid, mosaic, aneuploid, or undetermined. For the primary binary classification, embryos labeled euploid or aneuploid by PGT-A were used; mosaic and undetermined embryos were excluded. Imaging conditions were standardized as follows: 10× objective (NA 0.3), bright-field red LED illumination (635 nm), z-stack of 7 planes at 15 μm steps (the central plane was used for analysis), image resolution 800 × 800 pixels, and acquisition interval of 10 min.

The datasets were split into training, validation, and internal test sets in an 8:1:1 ratio, with stratified sampling to maintain proportional representation from both institutions. All dataset splits were performed at the patient level to ensure that embryos derived from the same patient were confined to a single data split, thereby preventing information leakage across training, validation, and test sets. The pregnancy dataset exhibited no substantial differences across data splits with respect to maternal age, inferred insemination method (IVF vs. ICSI), or clinical pregnancy rates (Supplementary Table S7). Similarly, the PGT-A dataset showed highly comparable clinical characteristics across training, validation, and test sets, including maternal age, inferred insemination method (IVF vs. ICSI), and PGT-A outcome distributions, indicating no substantial imbalance across data splits (Supplementary Table S8). Additionally, fully independent external test datasets were collected at two separate IVF centers in Japan—one for the Clinical Pregnancy Prediction Model (*n* = 112) and the other for the Ploidy Prediction Model (*n* = 157), between January 2019 and December 2021. These external datasets were obtained from institutions not involved in model training and followed identical inclusion criteria and outcome definitions. Basic clinical characteristics of the external test datasets are summarized in Supplementary Table S7 and S8. These datasets were reserved exclusively for external validation and were not used during any stage of model development.

### Embryo detection and image preprocessing

An overview of the entire pipeline, including the embryo detection and stabilization procedure, is illustrated in Supplementary Figure S4. Due to the nature of the embryo culture process—where multiple embryos are cultured simultaneously in a single dish—embryos often exhibit positional shifts within the video frames caused by manipulations of adjacent embryos rather than inherent embryo movement. To address this, we developed and trained a YOLOv5-based object detection model on a manually annotated subset of embryo images from our dataset, as publicly available pretrained models were not optimized for the unique visual characteristics of time-lapse embryo imaging. The detected embryo region in each frame was then used for precise centering within the video sequence. In addition, all cropped embryo regions were resized using a single fixed magnification factor chosen to ensure that the largest embryo size observed across frames was fully contained within the field of view; this procedure was applied uniformly to all embryos and all frames. This process effectively removed irrelevant background information and reduced positional variability, ensuring that the embryo consistently occupied the identical location across all frames. The detector achieved excellent performance on the held-out validation set (mAP@0.5 = 0.992, precision = 0.99, recall = 0.99), confirming reliable embryo detection for subsequent stabilization.

From a technical standpoint, such stabilization and background removal are critical for convolutional neural networks, as maintaining spatial consistency across frames reduces noise in feature extraction and allows the model to focus on biologically relevant morphological and dynamic patterns. By maintaining a stable field of view centered on the embryo, the preprocessing pipeline enhances the consistency and quality of the input data, directly contributing to improved model performance and interpretability.

### Model development

Two separate deep learning models were constructed: (1) a Clinical Pregnancy Prediction Model for gestational sac confirmation, and (2) a PGT-A-based Ploidy Prediction Model for euploid vs. aneuploid classification. Both models were implemented in PyTorch. We utilized the X3D-M architecture—a state-of-the-art spatiotemporal convolutional neural network developed by Facebook AI Research^27^—using the PyTorchVideo implementation maintained by Facebook AI Research. The X3D-M variant contains approximately 3.8 million trainable parameters, as confirmed by model inspection in PyTorch. The X3D-M backbone was initialized with weights pretrained on the Kinetics-400 dataset and subsequently fine-tuned end-to-end on the embryo time-lapse dataset.

Input features for both models consisted of preprocessed time-lapse embryo imaging and maternal age (as a continuous scalar feature). No post-outcome or future clinical information beyond the time point of each prediction was provided to the model. Each video was segmented into non-overlapping clips, each comprising 16 frames sampled at 5-frame intervals and resized from 800 × 800 to 400 × 400 pixels. The number of clips per video varied depending on the embryo stage at transfer, with a maximum of approximately 10 clips per embryo. The X3D-based model independently processed each clip to extract a deep video feature vector. Maternal age and the clip start time (elapsed hours post-fertilization, provided as a continuous scalar) were concatenated to the feature vector prior to the fully connected prediction layers, allowing both morphokinetic, temporal, and clinical information to contribute to the prediction. Maternal age was treated as a continuous scalar variable. Age was provided once per embryo as an auxiliary input alongside video-derived features.

For model training, the Adam optimizer (learning rate = 0.0001) was defined within the PyTorch Lightning module, with a batch size of 16 and cross-entropy loss. Training was performed on NVIDIA H100 GPUs, with a typical training time of approximately 6–8 h per model. We applied a dropout rate of 0.5 and early stopping based on validation loss, with a patience of 30 epochs and a maximum of 500 epochs; all models converged within 100 epochs. In practice, early stopping was typically triggered between 30 and 50 epochs, depending on the random initialization and dataset split. Data augmentation included random cropping (± 10%), horizontal flipping, brightness and contrast jittering (± 15%), and temporal jittering around clip boundaries. Augmentations that modify absolute developmental timing were intentionally avoided to prevent unintended temporal information leakage. Hyperparameters (learning rate, batch size, dropout, clip length, and sampling interval) were selected based on preliminary tuning experiments conducted on the validation set, prioritizing stability of training, avoidance of overfitting, and computational tractability. Cross-validation was not used; instead, a fixed train/validation/test split (8:1:1) was applied to maintain consistency across all ablation studies and ensemble models. Final models were evaluated on a held-out internal test set and on fully independent external datasets. These external datasets were never used for model development, hyperparameter selection, augmentation design, or early stopping, ensuring unbiased estimation of generalization performance. All architectural and training design choices were fixed prior to evaluation on the internal and external test sets.

### Ensemble method using robust Z-score

For each prediction task, we trained ten independently initialized models, each with different random seeds, to ensure diversity in learned parameters and reduce the risk of overfitting to a specific data partition. Since the output probability ranges of each model could differ (owing to stochastic optimization and inherent variability in deep learning), we applied a robust z-score normalization to the clip-level prediction scores prior to ensemble integration (Supplementary Figure S1).

For each embryo, the ten model predictions were first normalized as:

$$z_{i}=(x_{i}-median(x))/[1.4826\times MAD(x)]$$

where x_i is the prediction from the i-th model, median(x) is the median of the ten predictions, MAD(x) is the median absolute deviation, and 1.4826 is a scaling factor for standard normal approximation.

The ensemble prediction for each embryo was then computed as the median of the ten normalized scores, thereby reducing the influence of model-specific outliers and harmonizing the scale of predictions across models. Robust z-score normalization parameters were derived from the validation dataset and then fixed for use in the internal and external test datasets, ensuring consistent scaling and enabling robust comparison of prediction performance across independent cohorts. The ensemble size was fixed at 10 models a priori to balance variance reduction and computational cost, and no tuning of ensemble size was performed on the test or external datasets.

### Temporal evaluation

To capture the temporal dynamics of embryonic development, prediction scores were generated at multiple discrete time points corresponding to key preimplantation stages, as well as over cumulative intervals. These evaluations allowed us to assess not only the peak predictive performance but also the earliest time point at which clinically useful accuracy could be achieved. Predictive performance at each time point was evaluated using AUROC.

### Performance uncertainty (confidence intervals)

We estimated 95% confidence intervals (CIs) for AUROC using a non-parametric bootstrap procedure with 2000 resamples. For each resample, embryos were sampled with replacement from the evaluation cohort, and AUROC was recalculated; the 2.5th and 97.5th percentiles of the bootstrap distribution were used to define the 95% CI. Importantly, this bootstrap-based CI estimation was applied only to the final ensemble model, using the final ensemble prediction score per embryo, and is reported for the validation, internal test, and external test datasets (Fig. 1 and corresponding tables). In contrast, time-point and cumulative-window analyses summarized in Tables 1 and 2 report mean ± SD of AUROC across ten independently trained single models, and do not represent bootstrap-based confidence intervals.

### Statistical analysis

Model performance was evaluated using the AUROC across the validation, internal test, and external test datasets. For temporal evaluation, prediction scores were calculated at multiple discrete time points and cumulative time windows to examine the contribution of specific developmental stages to model performance. For threshold-based evaluation, cut-off values were determined in the validation dataset using the ROC criterion that minimizes the distance to the upper-left corner (0,1) and then applied unchanged to the internal and external test sets. Sensitivity, specificity, accuracy, and 95% confidence intervals were reported at these cut-offs.

## Supplementary Information

Below is the link to the electronic supplementary material.

Supplementary Material 1

Supplementary Material 2

Supplementary Material 3

Supplementary Material 4

Supplementary Material 5

## Data Availability

The datasets analyzed during the current study contain sensitive clinical imaging data and proprietary annotations. As such, they are not publicly available but may be shared by the corresponding author upon reasonable request and subject to institutional and ethical approvals.

## References

[1] Gardner, D. K., Lane, M., Stevens, J., Schlenker, T. & Schoolcraft, W. B. Blastocyst score affects implantation and pregnancy outcome: towards a single blastocyst transfer. *Fertil. Steril.* **73**, 1155–1158. 10.1016/s0015-0282(00)00518-5 (2000). 10856474 10.1016/s0015-0282(00)00518-5

[2] Gardner, D. K. & Schoolcraft, W. B. Culture and transfer of human blastocysts. *Curr. Opin. Obstet. Gynecol.* **11**, 307–311. 10.1097/00001703-199906000-00013 (1999). 10369209 10.1097/00001703-199906000-00013

[3] Alpha Scientists in Reproductive, M. & & Embryology, E. S. I. G. o. The Istanbul consensus workshop on embryo assessment: proceedings of an expert meeting. Hum Reprod 26, 1270–1283, (2011). 10.1093/humrep/der037 10.1093/humrep/der037 21502182

[4] Coticchio, G. et al. The Istanbul consensus update: a revised ESHRE/ALPHA consensus on oocyte and embryo static and dynamic morphological assessmentdagger,double dagger. *Hum. Reprod.* **40**, 989–1035. 10.1093/humrep/deaf021 (2025). 40288770 10.1093/humrep/deaf021 PMC12127515

[5] Gao, J. et al. The correlation between morphological parameters and the incidence of de novo chromosomal abnormalities in 3238 biopsied blastocysts. *J. Assist. Reprod. Genet.* **40**, 1089–1098. 10.1007/s10815-023-02780-5 (2023). 37058258 10.1007/s10815-023-02780-5 PMC10239399

[6] Ginstrom Ernstad, E. et al. Preimplantation genetic testing and child health: a national register-based study. *Hum. Reprod.* **38**, 739–750. 10.1093/humrep/dead021 (2023). 36749096 10.1093/humrep/dead021 PMC10068295

[7] Mastenbroek, S. et al. In vitro fertilization with preimplantation genetic screening. *N Engl. J. Med.* **357**, 9–17. 10.1056/NEJMoa067744 (2007). 17611204 10.1056/NEJMoa067744

[8] Zhao, J. et al. Metabolic Profiles of Offspring Born From Biopsied Embryos from Toddlerhood to Preschool Age. *J. Clin. Endocrinol. Metab.* **110**, e980–e991. 10.1210/clinem/dgae315 (2025). 38805186 10.1210/clinem/dgae315

[9] Chavez-Badiola, A., Flores-Saiffe-Farias, A., Mendizabal-Ruiz, G., Drakeley, A. J. & Cohen, J. Embryo Ranking Intelligent Classification Algorithm (ERICA): artificial intelligence clinical assistant predicting embryo ploidy and implantation. *Reprod. Biomed. Online*. **41**, 585–593. 10.1016/j.rbmo.2020.07.003 (2020). 32843306 10.1016/j.rbmo.2020.07.003

[10] Khosravi, P. et al. Deep learning enables robust assessment and selection of human blastocysts after in vitro fertilization. *NPJ Digit. Med.* **2**, 21. 10.1038/s41746-019-0096-y (2019). 31304368 10.1038/s41746-019-0096-y PMC6550169

[11] Diakiw, S. M. et al. Development of an artificial intelligence model for predicting the likelihood of human embryo euploidy based on blastocyst images from multiple imaging systems during IVF. *Hum. Reprod.* **37**, 1746–1759. 10.1093/humrep/deac131 (2022). 35674312 10.1093/humrep/deac131 PMC9340116

[12] Bormann, C. L. et al. Performance of a deep learning based neural network in the selection of human blastocysts for implantation. *Elife* **9**10.7554/eLife.55301 (2020). 10.7554/eLife.55301 PMC7527234 32930094

[13] Wong, C. C. et al. Non-invasive imaging of human embryos before embryonic genome activation predicts development to the blastocyst stage. *Nat. Biotechnol.* **28**, 1115–1121. 10.1038/nbt.1686 (2010). 20890283 10.1038/nbt.1686

[14] Meseguer, M. et al. The use of morphokinetics as a predictor of embryo implantation. *Hum. Reprod.* **26**, 2658–2671. 10.1093/humrep/der256 (2011). 21828117 10.1093/humrep/der256

[15] Basile, N. et al. The use of morphokinetics as a predictor of implantation: a multicentric study to define and validate an algorithm for embryo selection. *Hum. Reprod.* **30**, 276–283. 10.1093/humrep/deu331 (2015). 25527613 10.1093/humrep/deu331

[16] Lewis, N. et al. Morphokinetics of early equine embryo development in vitro using time-lapse imaging, and use in selecting blastocysts for transfer. *Reprod. Fertil. Dev.* **31**, 1851–1861. 10.1071/RD19225 (2019). 31634434 10.1071/RD19225

[17] Tran, D., Cooke, S., Illingworth, P. J. & Gardner, D. K. Deep learning as a predictive tool for fetal heart pregnancy following time-lapse incubation and blastocyst transfer. *Hum. Reprod.* **34**, 1011–1018. 10.1093/humrep/dez064 (2019). 31111884 10.1093/humrep/dez064 PMC6554189

[18] Berntsen, J., Rimestad, J., Lassen, J. T., Tran, D. & Kragh, M. F. Robust and generalizable embryo selection based on artificial intelligence and time-lapse image sequences. *PLoS One*. **17**, e0262661. 10.1371/journal.pone.0262661 (2022). 35108306 10.1371/journal.pone.0262661 PMC8809568

[19] Wang, J., Guo, Y., Zhang, N. & Li, T. Research progress of time-lapse imaging technology and embryonic development potential: A review. *Med. (Baltim).* **102**, e35203. 10.1097/MD.0000000000035203 (2023). 10.1097/MD.0000000000035203 PMC10519478 37746957

[20] VerMilyea, M. et al. Development of an artificial intelligence-based assessment model for prediction of embryo viability using static images captured by optical light microscopy during IVF. *Hum. Reprod.* **35**, 770–784. 10.1093/humrep/deaa013 (2020). 32240301 10.1093/humrep/deaa013 PMC7192535

[21] Rajendran, S. et al. Automatic Ploidy Prediction and Quality Assessment of Human Blastocyst Using Time-Lapse Imaging. *bioRxiv*10.1101/2023.08.31.555741 (2023). 39237547 10.1038/s41467-024-51823-7 PMC11377764

[22] Fenwick, J., Platteau, P., Murdoch, A. P. & Herbert, M. Time from insemination to first cleavage predicts developmental competence of human preimplantation embryos in vitro. *Hum. Reprod.* **17**, 407–412. 10.1093/humrep/17.2.407 (2002). 11821286 10.1093/humrep/17.2.407

[23] Fu, J. et al. The influence of early cleavage on embryo developmental potential and IVF/ICSI outcome. *J. Assist. Reprod. Genet.* **26**, 437–441. 10.1007/s10815-009-9342-6 (2009). 19789972 10.1007/s10815-009-9342-6 PMC2767488

[24] Sharma, A. et al. Predicting Cell Cleavage Timings from Time-Lapse Videos of Human Embryos. *Big Data Cogn. Comput.* **7**, 91 (2023).

[25] Bamford, T. et al. Morphological and morphokinetic associations with aneuploidy: a systematic review and meta-analysis. *Hum. Reprod. Update*. **28**, 656–686. 10.1093/humupd/dmac022 (2022). 35613016 10.1093/humupd/dmac022

[26] Rotem, O. et al. Visual interpretability of image-based classification models by generative latent space disentanglement applied to in vitro fertilization. *Nat. Commun.* **15**, 7390. 10.1038/s41467-024-51136-9 (2024). 39191720 10.1038/s41467-024-51136-9 PMC11349992

[27] Feichtenhofer, C. X3D: Expanding Architectures for Efficient Video Recognition. IEEE/CVF Conference on Computer Vision and Pattern Recognition (CVPR), 203–213, (2020). 10.1109/CVPR42600.2020.00028 (2020).

