# Supplementary figures and images for: AI prediction models based on time-lapse imaging for good embryos with implantation potential and euploidy
Source: Sci Rep. 2026 Feb 19;16:9864. doi: 10.1038/s41598-026-40917-5 (PMC13018220; doi:10.1038/s41598-026-40917-5)

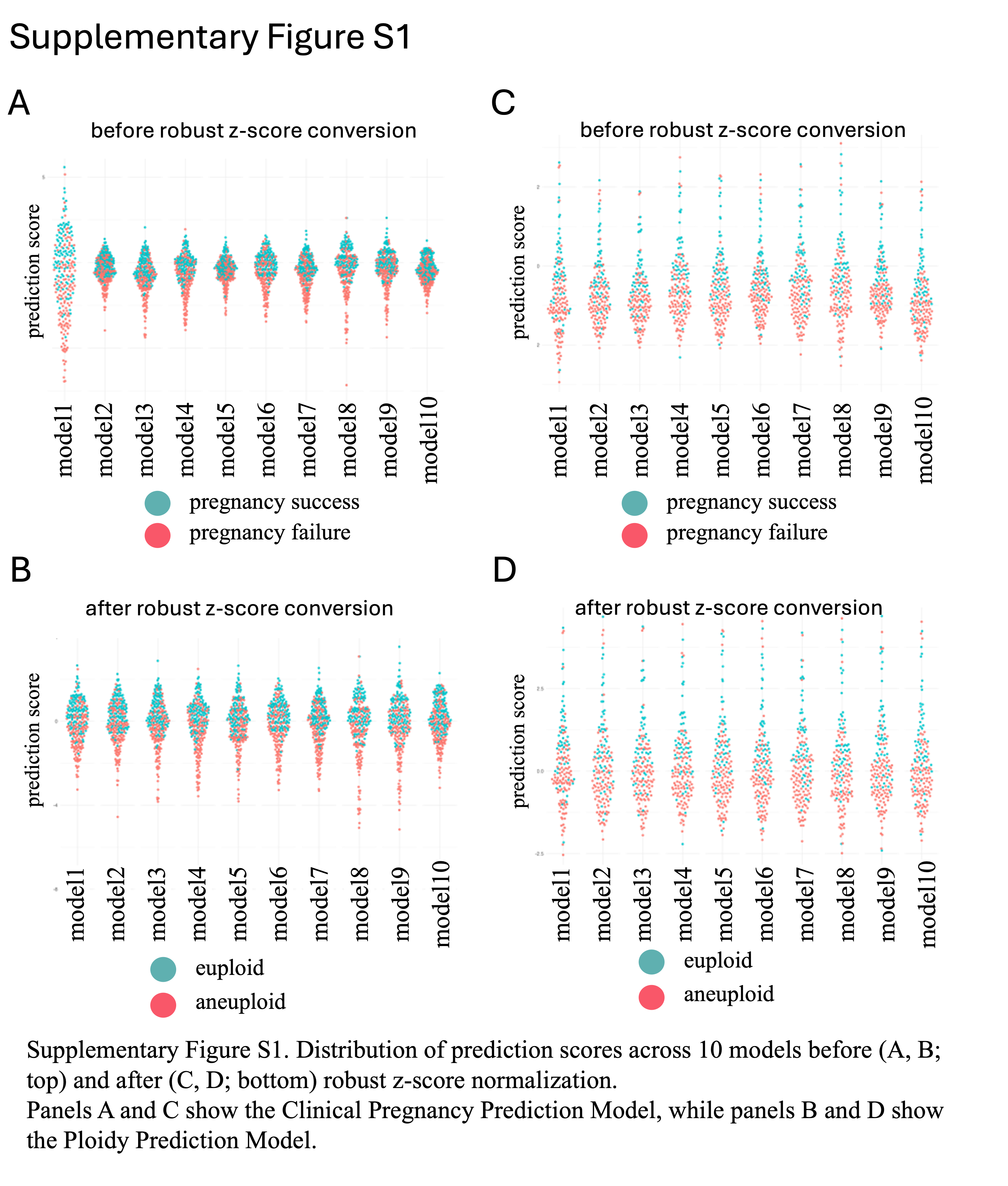

Supplement: Supplementary file 2 — Supplementary Material 2 [file 41598_2026_40917_MOESM2_ESM.tiff]

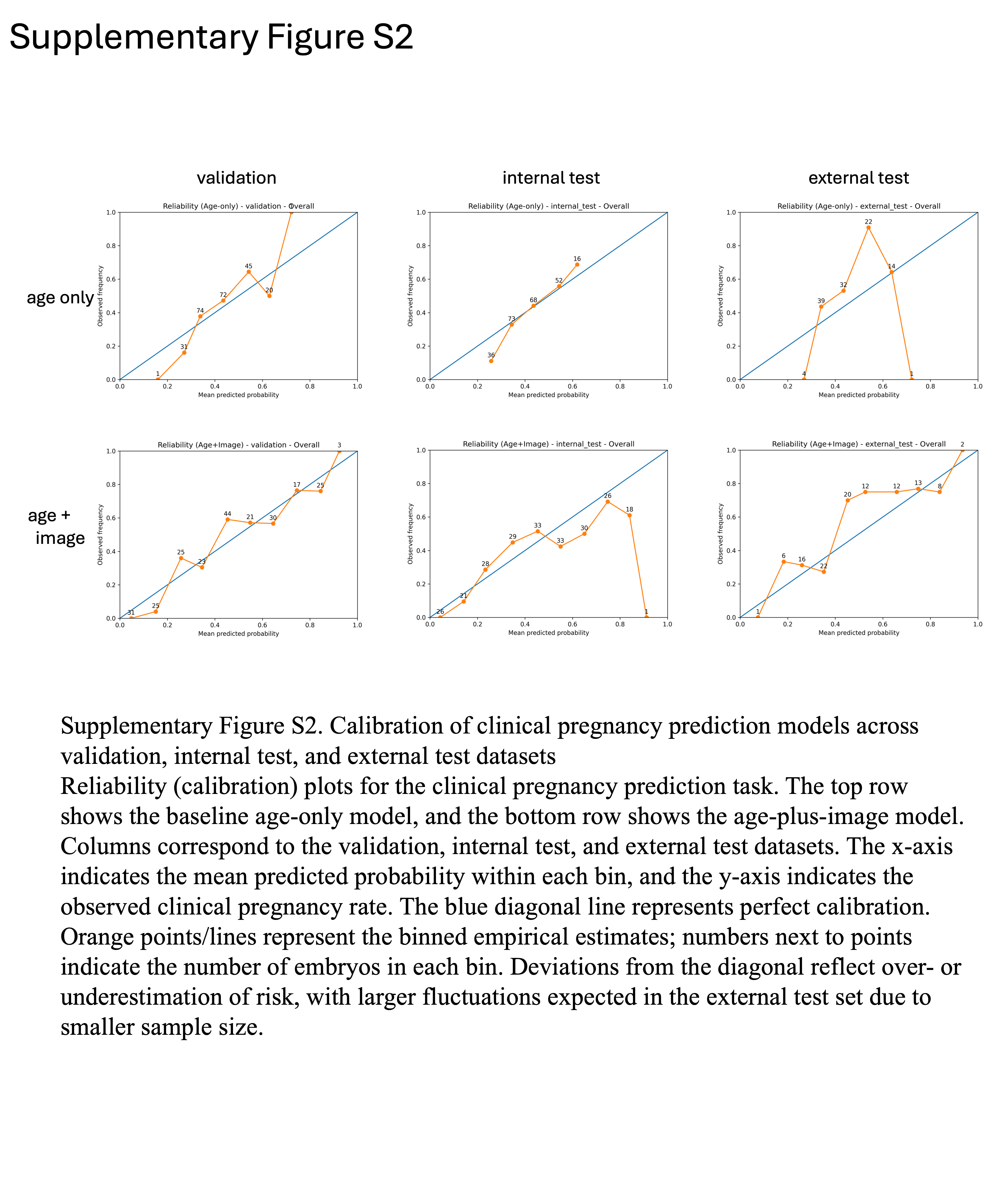

Supplement: Supplementary file 3 — Supplementary Material 3 [file 41598_2026_40917_MOESM3_ESM.tiff]

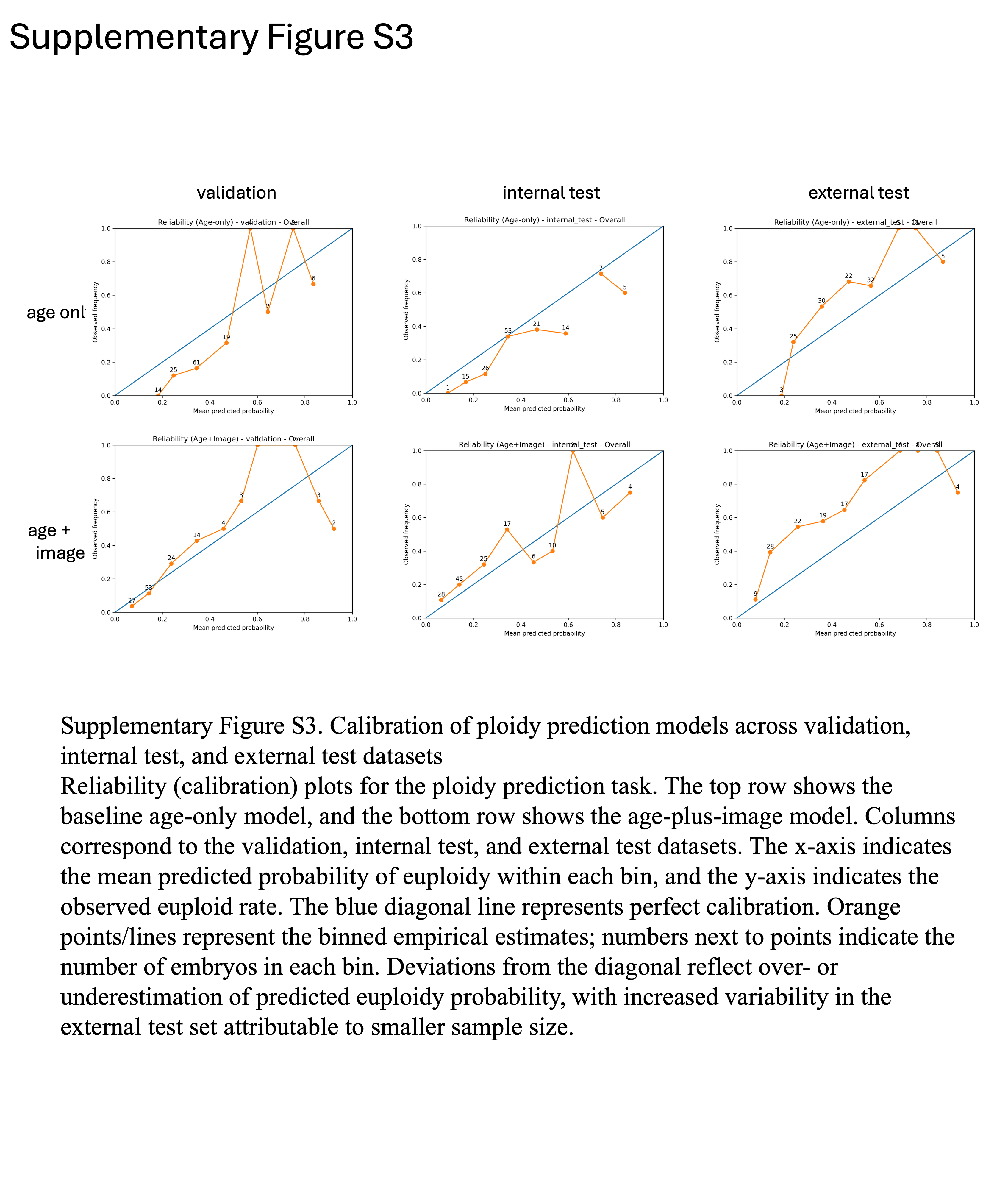

Supplement: Supplementary file 4 — Supplementary Material 4 [file 41598_2026_40917_MOESM4_ESM.tiff]

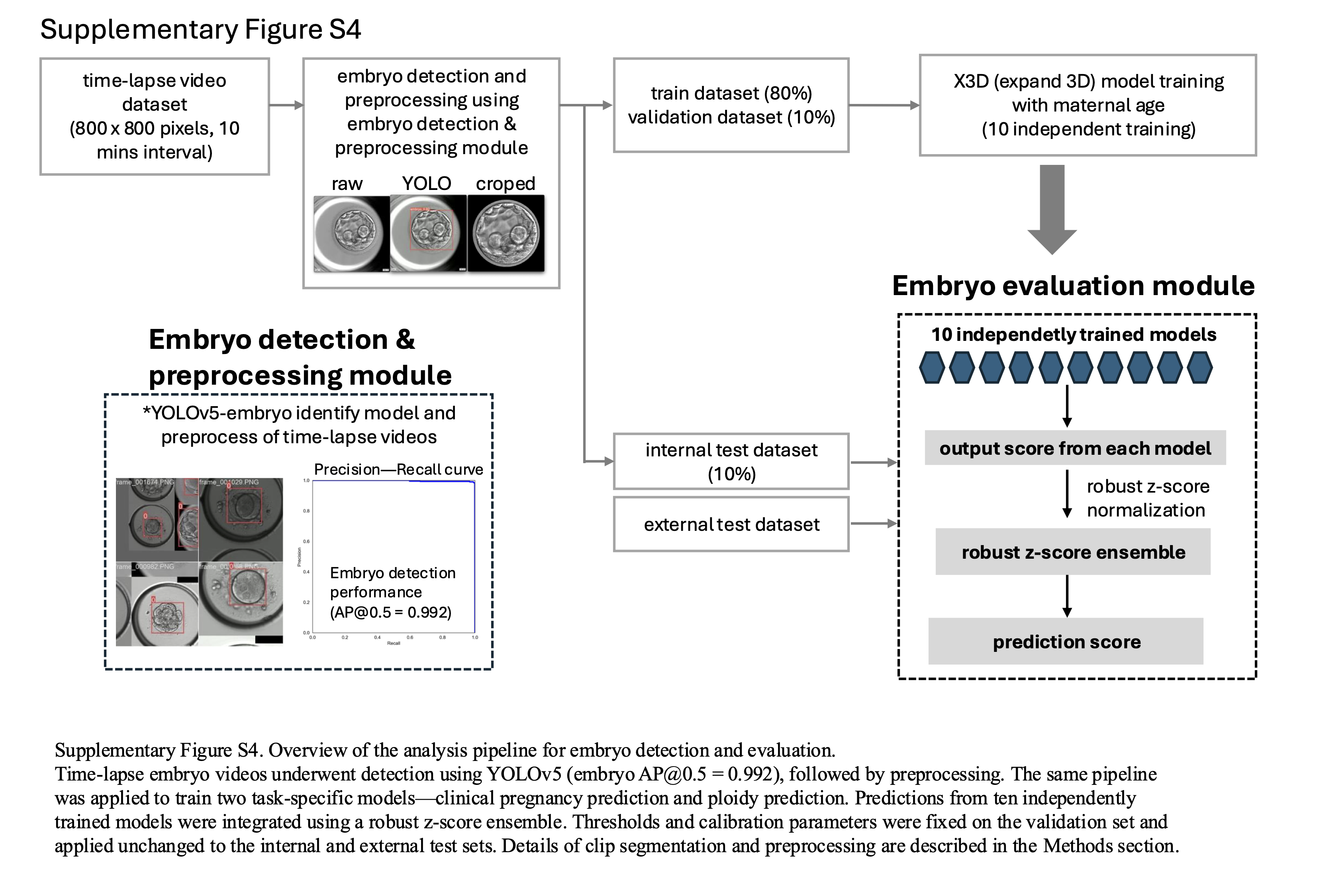

Supplement: Supplementary file 5 — Supplementary Material 5 [file 41598_2026_40917_MOESM5_ESM.tiff]
